# Single-Cell RNA Sequencing Reveals Cellular Heterogeneity and Developmental Dynamics of Goose Satellite Cells During Embryogenesis

**DOI:** 10.3390/cells15110983

**Published:** 2026-05-27

**Authors:** Cui Wang, Yi Liu, Guitao Jiang, Chuang Li, Kai Shi, Shufang Chen, Huiying Wang, Daqian He

**Affiliations:** 1 Institute of Animal Science and Veterinary Medicine, Shanghai Academy of Agricultural Sciences, Shanghai 201106, China; cuiwang518@saas.sh.cn (C.W.); liuyi20031194@163.com (Y.L.); huiyingwang@saas.sh.cn (H.W.); 2 Shanghai Municipal Key Laboratory of Agri-Genetics and Breeding, Shanghai 201106, China; 3 Yuelushan Laboratory, Changsha 410128, China; jiangguitao@hunaas.cn (G.J.); lichuang@hunaas.cn (C.L.); 4 Hunan Institute of Animal and Veterinary Science, Changsha 410128, China; 5 NingBo Academy of Agricultural Sciences, Ningbo 315040, China; shikai2024@126.com (K.S.); 13606780161@163.com (S.C.)

**Keywords:** scRNA-seq, satellite cells, developmental dynamics, molecular signatures, goose embryogenesis

## Abstract

**Highlights:**

1. Single-cell RNA-seq of goose embryonic leg muscles identifies three functional states of satellite cells (quiescent, activated, proliferative/differentiating), with the quiescent pool declining and activated pools expanding from E13 to E23.
2. Pseudotime analysis reveals a linear progression from quiescence to differentiation, marked by dynamic expression of PAX7, MYF5, and MYOD1 that reflects sequential activation and commitment.
3. State-specific molecular signatures and dynamically regulated genes (ECM–receptor interaction, Wnt signaling) provide a high-resolution transcriptional atlas for understanding avian satellite cell state transitions.
4. Quiescent satellite cells exhibit the most extensive intercellular signaling networks (e.g., FGFR, Ephrin, Collagen, CADM), which progressively diminish upon activation, offering potential targets for poultry genetic improvement and muscle regeneration.

**Abstract:**

Skeletal muscle satellite cells (SMSCs) are essential for embryonic myogenesis and postnatal muscle regeneration; however, their cellular heterogeneity and transcriptional dynamics during avian development remain largely unexplored. Here, we performed single-cell RNA sequencing (scRNA-seq) on 42,886 cells isolated from goose leg muscles across four embryonic stages (E13, E15, E18, and E23), with each stage comprising pooled tissues from four female embryos. Unbiased clustering resolved 22 transcriptionally distinct clusters representing six major cell types—satellite cells, myocytes, fibro-adipogenic progenitors, endothelial cells, immune cells, and Schwann cells—with satellite cells being the most abundant. Satellite cells were further subdivided into three functional states (quiescent, activated, and proliferative/differentiating), which followed a continuous, linear pseudotime trajectory from early to late embryonic stages. This trajectory was marked by a progressive downregulation of stemness-associated regulators (e.g., *PAX7*) and upregulation of myogenic commitment and differentiation factors (e.g., *MYF5*, *MYOD1*, and *MYOG*), faithfully mirroring chronological development. Cell–cell communication analysis revealed that quiescent satellite cells exhibited the most extensive intercellular signaling networks (e.g., FGFR, Ephrin, collagen, CADM), whereas activated and proliferative/differentiating cells showed progressively diminished communication capacity. Across developmental stages, the contribution intensities of key signaling pathways—including SEMA6, CDH, FGF, LAMININ, MK, MPZ, CADM, FN1, and COLLAGEN—varied significantly among satellite cell states, indicating state-specific responsiveness to microenvironmental cues. Collectively, these findings demonstrate that satellite cells dynamically coordinate extrinsic signal integration with intrinsic differentiation programs to achieve orderly myogenic progression. This study provides a high-resolution single-cell atlas of goose SMSC development, uncovering subpopulation heterogeneity, state-specific molecular signatures, and key signaling pathways, with important implications for avian muscle biology and genetic improvement of poultry.

## 1. Introduction

Skeletal muscle is a crucial organ for locomotion and energy metabolism, comprising approximately 30–40% of the total mass in livestock and poultry [1]. Its development and postnatal growth directly determine key economic traits in animal production, including meat yield and quality. Myofibers, the fundamental contractile units of skeletal muscle, are primarily formed during embryogenesis through both hyperplastic and hypertrophic processes. Importantly, the final number of myofibers is largely established before birth, underscoring the pivotal role of embryonic development in determining ultimate muscle mass and meat production [2]. The formation and postnatal maintenance of myofibers are tightly regulated by a population of muscle-specific stem cells known as skeletal muscle satellite cells (SMSCs) [3].

SMSCs are responsible for postnatal muscle growth, regeneration, and maintenance. They serve as the principal source of myogenic progenitors during embryonic muscle formation, driving the hyperplastic phase [3]. Upon activation, SMSCs express myogenic regulatory factors (MRFs), such as *MYOD1* and *MYOG*, and subsequently differentiate into myoblasts and myocytes [4]. Thus, SMSCs represent an invaluable model for dissecting the molecular mechanisms that govern muscle development and meat quality in agricultural species.

Recent evidence indicates that SMSCs are not a homogeneous population but exhibit marked functional heterogeneity linked to distinct embryonic origins and regenerative capabilities [5,6]. For example, single-cell transcriptomic analyses in turkeys have revealed that satellite cell clones with different proliferation rates possess distinct gene expression profiles: fast-growing clones are enriched in muscle development genes, whereas slow-growing clones show enrichment for extracellular matrix and cell–cell communication genes [7]. Our previous studies on goose SMSCs using bulk transcriptomic and metabolomic approaches identified the PPAR signaling pathway—along with key regulators such as PPARG and IGF1—as central to differentiation, characterized by dynamic shifts in lipid metabolism and myogenesis [8]. Furthermore, transcriptomic atlases of embryonic goose SMSCs revealed stage-specific gene expression programs progressing from early myogenesis to later metabolic maturation and uncovered core regulatory networks involving factors such as *MEF2C* and *MYOD1* [9]. However, these population-level analyses inevitably mask underlying cellular heterogeneity, continuous differentiation trajectories, and intricate cell–cell communication networks within the SMSC pool, representing a significant knowledge gap.

Among poultry species, geese hold particular economic importance, especially in Asia and Europe, where they are valued for high meat yield, distinctive meat quality (e.g., favorable fatty acid composition and texture), and adaptability to extensive farming systems. Compared with chickens and ducks, geese exhibit unique muscle growth characteristics, including a longer embryonic development period and distinct patterns of muscle fiber hyperplasia and hypertrophy, which may contribute to their superior meat quality traits. Nevertheless, the cellular and molecular mechanisms underlying goose muscle development remain poorly understood, largely due to a lack of high-resolution transcriptomic data. Consequently, a systematic characterization of goose SMSCs at single-cell resolution is urgently needed to bridge this gap.

Single-cell RNA sequencing (scRNA-seq) has emerged as a transformative technology that enables the dissection of cellular diversity, definition of dynamic cellular states, and reconstruction of developmental lineages at unprecedented resolution. This approach has been successfully applied to unravel myogenic heterogeneity across several species. In chickens, single-nucleus RNA sequencing revealed extensive myonuclear heterogeneity and identified RBM24 as a key regulator of myoblast differentiation [10]. Similarly, scRNA-seq of embryonic chicken skeletal muscle uncovered distinct developmental trajectories for myoblasts and fibro-adipogenic progenitors across different breeds [11]. In cattle, scRNA-seq demonstrated that cultured satellite cells consist of subpopulations with divergent transcriptional and myogenic states, including fibro-adipogenic progenitors [12]. In humans, studies combining engineered 3D skeletal muscle models and scRNA-seq identified transcriptionally distinct quiescent and activated satellite cell subpopulations, with Notch and Wnt signaling pathways governing quiescence maintenance [13]. More recently, advances in goose scRNA-seq have begun to reveal dynamic changes in breast muscle cells during the embryonic development of Ding’an goose [14]. In parallel, a very recent study employed single-cell transcriptomics to investigate skeletal muscle differentiation across duck embryonic development, revealing cellular heterogeneity and dynamic transcriptional programs underlying myogenesis and key transitions in myofiber type specification [15]. Despite these across-species advances, a comprehensive, systematic characterization of SMSCs at single-cell resolution throughout avian embryogenesis—particularly in geese—has remained lacking.

Beyond cellular heterogeneity, emerging evidence highlights the critical role of cell–cell communication in coordinating myogenic progression. During skeletal muscle development, SMSCs do not act in isolation; instead, they continuously exchange signals with neighboring cell types, including myoblasts, fibro-adipogenic progenitors (FAPs), and immune cells, through ligand–receptor interactions. These communication networks regulate key processes such as quiescence maintenance, activation, proliferation, and differentiation. For example, Notch and Wnt signaling pathways mediate crosstalk between quiescent and activated satellite cells [16,17], whereas growth factor pathways such as IGF and FGF regulate myoblast fusion and myofiber maturation [18]. Disruption of these communication networks leads to impaired muscle regeneration and aberrant development [19]. Therefore, delineating cell–cell communication networks within the SMSC pool is essential for understanding how myogenic progression is orchestrated at the tissue level. However, such analyses remain unexplored in goose SMSCs.

To address these gaps and advance knowledge of goose muscle development, we first performed scRNA-seq analysis of SMSCs isolated from goose leg muscles at four embryonic stages (E13, E15, E18, and E23). This study aims to (1) characterize the cellular heterogeneity and subpopulation composition of goose SMSCs during embryogenesis; (2) reconstruct the continuous differentiation trajectory from quiescent to activated and proliferative/differentiating states; (3) identify state-specific gene expression dynamics and key regulatory signaling pathways; and (4) delineate cell–cell communication networks orchestrating myogenic progression. Our findings provide novel insights into the cellular basis of goose muscle development and establish a single-cell resource to inform precision breeding strategies aimed at enhancing poultry muscle growth and meat quality.

## 2. Materials and Methods

### 2.1. Tissue Collection and SMSC Purification

A total of 100 fertilized Zhedong White goose eggs were obtained from the Wenjie Goose Breeding Department of Xiangshan Co., Ltd. (Ningbo, China) and incubated in a standard commercial incubator (Zhonglian, China) at 37.5 °C with 60% relative humidity. Leg muscle tissues were collected from female embryos at embryonic days 13, 15, 18, and 23 (E13, E15, E18, and E23). For each developmental stage, leg muscle tissues were collected from four healthy female embryos, and tissues from the four embryos were pooled prior to satellite cell isolation. Embryonic sex was determined via *CHD1* gene primers as previously described [8]. Following approved protocols (IACUC of Shanghai Academy of Agricultural Sciences, SAASPZ0526168, and ARRIVE guidelines), embryos were euthanized by CO_2_ inhalation followed immediately by decapitation.

Satellite cells were isolated and purified according to our established protocol [9]. Briefly, muscle tissues were minced and digested in high-glucose DMEM with 2 mg/mL Dispase II and 4 mg/mL Collagenase II (Roche, Basel, Switzerland) at 37 °C for 50 min. Digestion was stopped using DMEM with 10% FBS. The cell suspension was filtered through a 70 μm strainer, centrifuged at 350× *g* for 8 min, and red blood cells were lysed with ACK buffer. Differential plating was performed twice to enrich SMSCs, exploiting fibroblast attachment for one hour each time. Purity was confirmed by Pax7 immunofluorescence staining [9]. Enriched SMSCs were cryopreserved in liquid nitrogen for subsequent scRNA-seq.

### 2.2. scRNA-Seq Library Construction and Sequencing

Thawed SMSCs were filtered through a 40 μm strainer, centrifuged at 300× *g* for 5 min at 4 °C, and resuspended in PBS with 0.04% BSA. Only samples with >85% viability (trypan blue exclusion) and 400–1200 cells/μL were processed for single-cell capture. A Chromium Single-Cell 3′ Library Kit (10× Genomics, Pleasanton, CA, USA) was used for cell capture, barcoding, and cDNA synthesis following the manufacturer’s protocol [20]. Libraries were quantified using a Bioanalyzer 2100 and Qubit, then sequenced on Illumina NovaSeq platforms Shanghai Majorbio Bio-pharm Technology Co., Ltd. (Shanghai, China).

### 2.3. Single-Cell RNA-Seq Data Processing and Analysis

Raw FASTQ data were processed with Cell Ranger (v7.1.0) and aligned to the goose reference genome (GCF_002166845.1) using STAR [11]. The gene–barcode matrix was analyzed with Seurat (v5.3.1) [21]. Quality filtering removed cells with <300 or >6500 genes, <1000 or >50,000 UMIs, or >20% mitochondrial reads (mitochondrial genes annotated from the Bean goose genome) [22]. Doublets were excluded using scDblFinder (v1.12.0+) [23]. Data were normalized and scaled, regressing out mitochondrial content.

Integration across samples was performed with FindIntegrationAnchors and IntegrateData [21,24], with further batch correction using Harmony [25]. Principal component analysis (PCA, top 30 PCs) was followed by graph-based clustering (resolution 0.5–1.2), and visualization by UMAP and t-SNE [26]. Differential expression was assessed using the Wilcoxon rank-sum test (adjusted *p* < 0.05).

### 2.4. Cell Clustering and Cell Type Identification

To characterize cellular heterogeneity, unsupervised clustering was performed using the Louvain algorithm on a shared nearest neighbor (SNN) graph constructed from PCA-reduced dimensions [21]. The resolution parameter was optimized to generate stable and biologically meaningful clusters.

For each cluster, differentially expressed genes (markers) were identified using the FindAllMarkers function with the following thresholds: log-fold change ≥ 0.05, minimum percentage of expression in either group (min.pct) ≥ 0.1, and minimum absolute expression difference between groups (min.diff.pct) ≥ 0.05. Genes meeting all criteria were retained as candidate markers.

Cell type annotation followed a multi-step integrative approach. First, a preliminary assignment was performed manually based on well-established marker genes from the literature [27,28,29]. Second, two computational methods—SCINA (a semi-supervised algorithm using predefined marker sets) and AddModuleScore (calculating average expression of gene sets per cell)—were applied to refine and cross-validate annotations [30]. Third, Gene Ontology (GO) enrichment analysis was performed on cluster-specific markers to confirm biological relevance [31]. The final cell type labels were generated by integrating the results from manual annotation, computational assignment, and functional enrichment.

### 2.5. Differential Expression Analysis and Functional Enrichment

Differential expression genes (DEGs) between clusters or samples were identified using Seurat’s FindMarkers function (likelihood ratio test; |log2FC| ≥ 0.25, Q < 0.05). Gene Ontology (GO) terms enrichment was analyzed using topGO (v2.50.0), and Kyoto Encyclopedia of Genes and Genomes (KEGG) pathway enrichment analysis was per formed using clusterProfiler (v4.6.0). A protein–protein interaction (PPI) network was constructed using the STRING database to identify interactions among DEGs, and the resulting network was visualized with Cytoscape software (v3.8.0) [32].

### 2.6. Pseudotime Analysis of SMSCs

To resolve SMSC developmental trajectories and transcriptional dynamics, pseudotime analysis was performed using Monocle2 and Monocle3 [33,34]. SMSCs were extracted from Seurat objects, reprocessed (log-normalization, feature selection, PCA), and converted to Monocle cell_data_set objects, aligning on UMAP embeddings for consistency. Trajectories were constructed using learn_graph, with order_cells assigning pseudotime states. Monocle2 was run in parallel to validate Monocle3 results. This approach provided comprehensive differentiation landscape characterization and identified lineage branching points.

### 2.7. Cell–Cell Communication Analysis

Cell–cell communication among populations was analyzed with CellChat (v1.6.1) [35], which applies a mass-action model integrating curated ligand-receptor pairs (CellChatDB). Significant interactions were selected via permutation testing. Signaling networks were visualized with circle, bubble, and role heatmaps (as sender, receiver, mediator, or influencer) [35,36]. This enabled exploration of SMSC-driven signaling pathways within the cellular microenvironment.

## 3. Results

### 3.1. Single-Cell Transcriptome Profiling Reveals Cellular Heterogeneity and Dynamic Composition of Satellite Cells During Embryonic Leg Muscle Development

**Figure 1:**
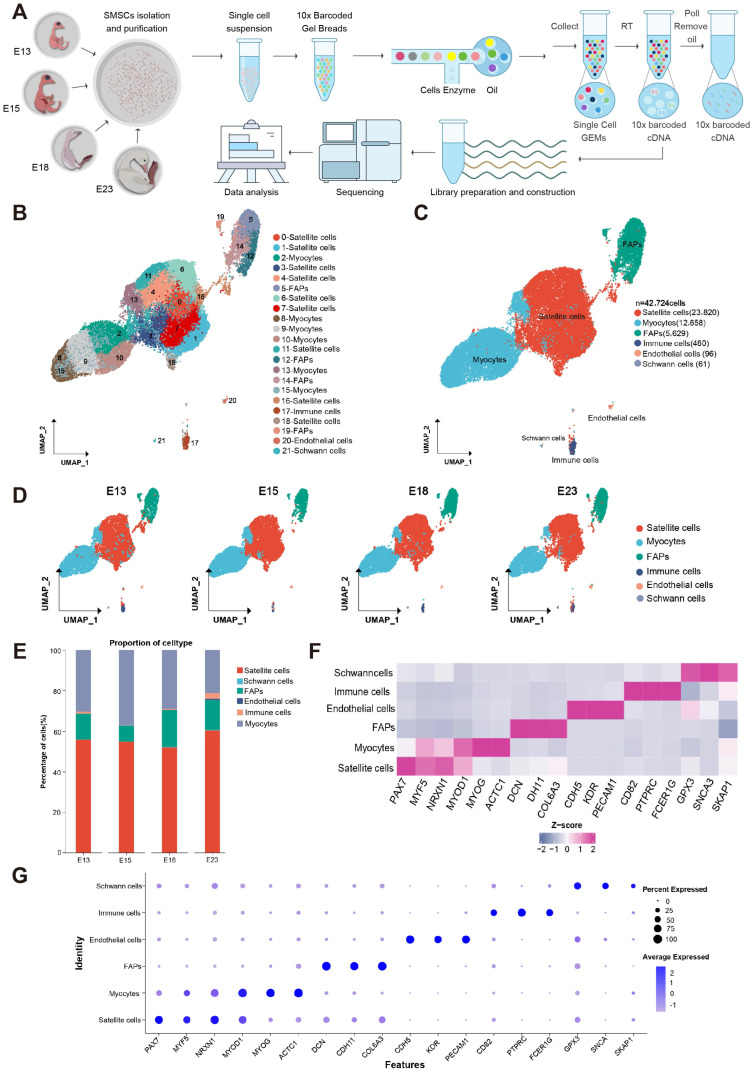
Experimental workflow and cell type identification during embryonic skeletal muscle development: (**A**) Schematic overview of the single-cell isolation, 10× Genomics library preparation, and data analysis pipeline. (**B**) Cellular composition of identified cell types (n = 42,724 cells). (**C**) UMAP visualization of major cell types. (**D**) UMAP projection of satellite cells across embryonic stages (E13, E15, E18, and E23). (**E**) Relative proportions of major cell types at each developmental stage. (**F**) Heatmap showing marker gene expression across major cell populations. (**G**) Dot plot showing expression of canonical marker genes in each cell type.

To comprehensively characterize the cellular landscape underlying goose SMSC development, we performed 10x Genomics scRNA-seq on SMSCs isolated from the leg muscle tissues of ZW geese at four key embryonic stages: E13, E15, E18, and E23 (Figure 1A). Following rigorous quality control, a total of 42,886 cells were retained for analysis, distributed as follows: 13,346 cells from E13, 10,048 from E15, 9780 from E18, and 9712 from E23. For all four libraries, the mean number of reads per cell exceeded 25,000, and the median number of genes detected per cell was over 3100 (Table 1), indicating robust data and quality suitable for downstream analyses.

Unsupervised Uniform Manifold Approximation and Projection (UMAP) analysis of 42,724 high-quality cells revealed 22 transcriptionally distinct clusters (Figure 1B). Based on differential gene expression and canonical marker genes, six major cell populations were annotated: satellite cells (clusters 0, 1, 3, 4, 6, 7, 11, 16, and 18), myocytes (clusters 2, 8, 9, 10, 13, and 15), FAPs (clusters 5, 12, 14, and 19), endothelial cells (cluster 20), immune cells (cluster 17), and Schwann cells (cluster 21) (Figure 1C). Among these, satellite cells were most abundant (23,820 cells), followed by myocytes (12,658) and FAPs (5629), with the remaining minor populations comprising immune (460), endothelial (96), and Schwann cells (61). All six cell types were consistently detected across all stages, confirming dataset reliability (Figure 1D), though their proportions varied during embryogenesis (Figure 1E; Table S1).

Distinct cell populations exhibited highly specific marker gene expression signatures (Figure 1F,G). Satellite cells demonstrated high expression of *PAX7*, *MYF5*, and *NRXN1*; myocytes were marked by *MYOG*, *ACTC1*, and *MYOD1*; and FAPs by *DCN*, *CDH11*, and *COL6A3*. Additional markers were detected in endothelial cells (*KDR*, *PECAM1*), immune cells (*CD82*, *PTPRC*, *FCER1G*), and Schwann cells (*GPX3*, *SNCA*, *SKAP1*). These expression patterns not only validated cell type assignments but also highlighted the establishment and maintenance of transcriptionally discrete identities during goose embryonic leg muscle development. These data provide a solid molecular framework to investigate the specialization and interaction of distinct cell types during muscle development.

Notably, both satellite cells and myocytes exhibited overlapping expression of key myogenic factors, including *MYF5*, *NRXN1*, and *MYOD1* (Figure 1F,G), whereas these genes were absent in other cell types (FAPs, endothelial, immune, and Schwann cells). This shared transcriptional program suggests a continuous differentiation trajectory from satellite cells toward myocytes, involving gradual transitions in gene expression rather than abrupt switches. The detection of *MYOD1* in a subset of satellite cells likely reflects activated or early-committed myogenic progenitors, whereas the expression of *NRXN1* may indicate previously unrecognized roles in mediating cell–cell interactions during muscle morphogenesis. Collectively, these findings further support the validity of our cell type annotations and underscore the transitional continuum of myogenic progression in goose embryonic skeletal muscle.

### 3.2. Dissecting Satellite Cell Heterogeneity and Dynamics During Myogenic Progression

**Figure 2:**
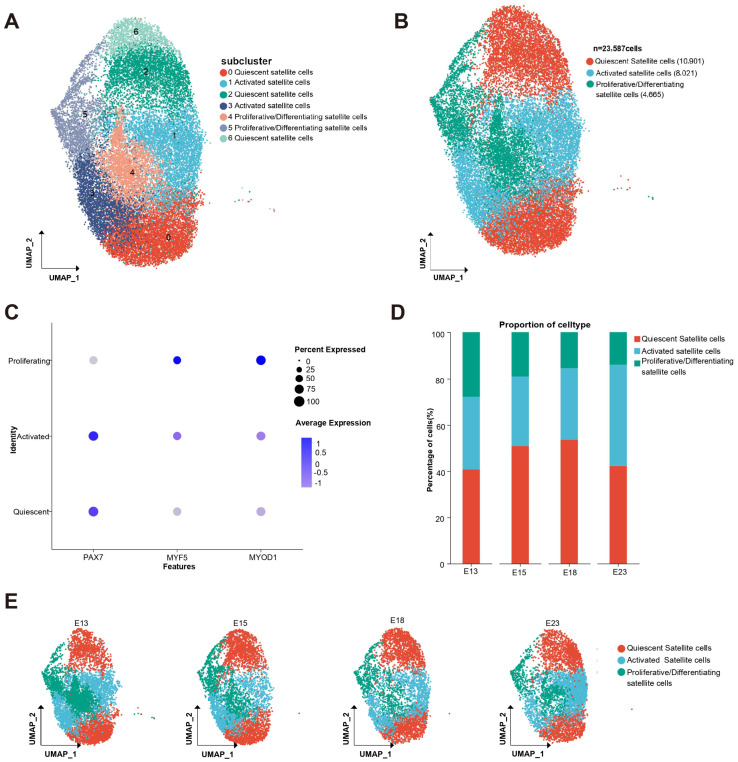
Heterogeneity of satellite cells during embryonic skeletal muscle development: (**A**) UMAP visualization showing satellite cell subclusters (0–6). (**B**) UMAP projection of the three major functional states: quiescent, activated, and proliferative/differentiating satellite cells. (**C**) Dot plot showing expression of the canonical markers *PAX7*, *MYF5*, and *MYOD1* across subclusters. (**D**) Bar plot showing the relative proportions of the three cell types at each developmental stage (E13, E15, E18, and E23). (**E**) UMAP projection of satellite cells colored by embryonic stage (E13 to E23).

To explore the transcriptional heterogeneity of satellite cells during embryonic skeletal muscle development, we performed UMAP-based dimensionality reduction and unsupervised clustering on 23,587 satellite cells isolated from the E13, E15, E18, and E23 stages. This analysis identified seven distinct subclusters (clusters 0–6; Figure 2A).

By integrating differential gene expression patterns and established marker genes—including the quiescence-associated factor *PAX7*, the early activation marker *MYF5*, and the myogenic commitment gene *MYOD1* (Figure 2B)—we annotated these subclusters into three main functional states (Figure 2C): quiescent satellite cells (clusters 0, 2, 6), activated satellite cells (clusters 1, 3), and proliferative/differentiating satellite cells (clusters 4, 5).

Among all satellite cells, quiescent cells accounted for the largest proportion (10,901 cells; ~46.2%), followed by activated cells (8021; ~34.0%) and proliferative/differentiating cells (4665; ~19.8%) (Figure 2B). These three functional states were present across all examined embryonic stages (E13, E15, E18, and E23; Figure 2D,E), indicating that transitions between quiescence, activation, and proliferation are continuous and tightly regulated throughout muscle development.

Dynamic changes in subpopulation proportions were observed during development (Figure 2D,E). Proliferative/differentiating satellite cells were more abundant at the early stages (E13–E15), corresponding to active muscle growth and myofiber formation. Conversely, the proportion of quiescent satellite cells increased at later stages (E18–E23), reflecting the progressive establishment of a quiescent stem cell pool poised for postnatal muscle regeneration. This developmental transition was further supported by the dynamic expression profiles of key regulatory genes (Figure 2B): whereas *PAX7* was broadly expressed across all subsets, *MYF5* and *MYOD1* were predominantly upregulated in activated and proliferative/differentiating cells, consistent with their roles in myogenic progression.

Collectively, these data provide a high-resolution transcriptional atlas of satellite cell heterogeneity during embryonic myogenesis, revealing that the quiescent state itself comprises multiple transcriptionally distinct subpopulations and that the shift from proliferation to quiescence is underpinned by dynamic regulation of key myogenic factors.

### 3.3. Identification of Key Differentially Expressed Genes Underlying Satellite Cell Development

**Figure 3:**
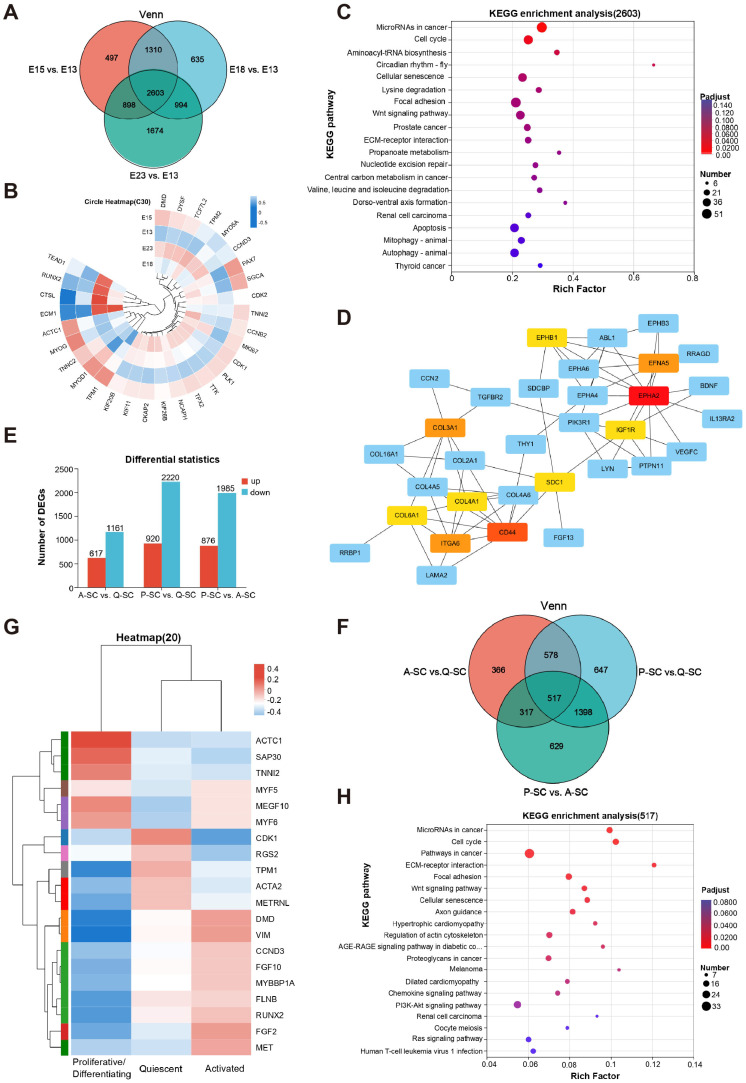
Differential gene expression and functional enrichment during satellite cell development: (**A**) Venn diagram showing DEGs across embryonic stages. (**B**) Circular heatmap of 30 key DEGs across satellite cell subpopulations. (**C**) KEGG enrichment analysis of the 2603 DEGs. (**D**) Protein–protein interaction (PPI) network of core DEGs shared across E13, E15, E18, and E23 during satellite cell development. (**E**) Bar plot showing the number of DEGs between each pair of adult satellite cell subpopulations. (**F**) Venn diagram of DEGs across three satellite cell subpopulations. (**G**) Heatmap of 20 key DEGs across three satellite cell subpopulations. The left panel shows a gene clustering dendrogram, and the right panel lists the gene names. The closer two gene branches to each other, the more similar their expression levels. (**H**) KEGG enrichment analysis of the 517 shared DEGs.

To elucidate the transcriptomic changes driving satellite cell development during goose embryogenesis, we performed differential gene expression analysis (|log_2_FC| > 1, adjusted *p* < 0.05) across E13, E15, E18, and E23 stages. Overlapping analysis (Figure 3A) identified 1310 DEGs common to E15 vs. E13 and E18 vs. E13, 994 genes common between E15 vs. E13 and E23 vs. E13, and 898 shared between E18 vs. E13 and E23 vs. E13. Importantly, 2603 DEGs were consistently altered in all three pairwise comparisons, representing core transcriptional regulators of satellite cell progression (Figure 3A, Table S1).

Functional categorization of these 2603 DEGs highlighted numerous hallmarks of satellite cell biology (Figure 3B). These included transcription factors critical for myogenic progression (*PAX7* for stemness, *MYOD1* for commitment, *MYOG* for differentiation), as well as *RUNX2*, *TEAD1*, and *TCF7L2*, which integrate developmental and signaling cues. Genes involved in cell cycle regulation (*MKI67*, *NCAPH*, *CDK1/2*, *CCNB2*, *CCND3*, *TTK*, *PLK1*, *KIF* family, *TPX2*, and *CKAP2*) and in sarcomeric or cytoskeletal structure (*ACTC1*, *TPM1/2*, *TNNI2*, *TNNC2*, *DMD*, *DYSF*, *SGCA*, and *MYO5A*) were also upregulated, along with metabolic and ECM remodeling factors (*ECM1*, *CTSL*). The concerted upregulation of these genes suggests a developmental program wherein satellite cells transition from quiescence through activation and expansion to terminal differentiation and myofiber assembly.

Gene Ontology (GO) enrichment analysis revealed that the core DEGs are predominantly localized to the nucleus and organelle membranes, where they are involved in transcriptional regulation, cellular biosynthesis, and cytoskeletal and sarcomere organization. Their molecular functions were enriched for organic cyclic compound and nucleoside phosphate binding (Figure S1, Table S2). More notably, KEGG pathway analysis further demonstrated significant enrichment for cell cycle control, metabolic reprogramming, and cell fate decisions (e.g., apoptosis, autophagy, and senescence), as well as canonical developmental pathways such as Wnt signaling and ECM–receptor interaction (Figure 3C, Table S3). These findings implicate precise regulation of proliferation, metabolic adaptation, and microenvironmental signaling in satellite cell fate transitions and muscle development.

Next, we constructed a protein–protein interaction (PPI) network based on the 2603 core differentially expressed genes. The PPI network revealed that collagen family members (e.g., COL1A1, COL1A2, COL3A1, COL6A1) serve as central nodes, forming highly interconnected clusters (Figure 3D). These collagen genes not only exhibit dense interconnections among themselves but also show close associations with various extracellular matrix (ECM)-related proteins (e.g., LAMA2, COL4A1, COL4A5), integrin signaling molecules (ITGA6, CD44), and cytoskeletal and signal regulators (e.g., TGFBR2, PIK3R1, ABL1, EPHA2, EPHB1, SDC1, THY1, PTPN11, LYN). These interactions suggest a close regulatory link between ECM remodeling and myofiber formation during goose embryonic satellite cell development. Nodes with high connectivity (degree), such as COL1A1, COL1A2, CD44, and TGFBR2, may serve as potential key driver genes governing satellite cell fate determination and muscle tissue construction. In summary, the PPI network analysis not only validates the findings of the preceding KEGG pathway enrichment analysis regarding ECM–receptor interaction and focal adhesion pathways but also suggests that the collagen family may play dual roles in satellite cell development, providing both structural support and signal regulation.

To further delineate transcriptional heterogeneity, we compared gene expression among quiescent (Q-SC), activated (A-SC), and proliferative/differentiating (P-SC) satellite cell subpopulations. Differential expression analysis revealed 617 upregulated and 1161 downregulated genes in A-SC vs. Q-SC; 920 upregulated and 2220 downregulated in P-SC vs. Q-SC; and 876 upregulated and 1985 downregulated in P-SC vs. A-SC. Notably, 517 DEGs were shared across all pairwise comparisons (Figure 3F; Table S4).

These common DEGs encompassed genes essential for muscle contraction (*ACTA2*, *ACTC1*, *TPM1*, *DMD*), cell cycle regulation (*CDK1*, *CCND3*), growth signaling (*FGFR1*, *FGFR2*, *MET*), myogenic differentiation (*MYF5*, *MYF6*, *RUNX2*, *SAP30*), and cytoskeletal remodeling (*VIM*, *FLNB*) (Figure 3G). Functional enrichment analysis showed prominent involvement in cell cycle regulation, cancer pathways, ECM–receptor interaction, Wnt signaling, focal adhesion, and PI3K-Akt signaling (Figure 3H; Table S5), highlighting the functional importance of these genes in regulating muscle cell proliferation, differentiation, adhesion, and structure. The significant enrichment for cancer-related pathways also raises the possibility of shared signaling mechanisms between muscle development and tumorigenesis, warranting further investigation.

### 3.4. Pseudotime Trajectory Analysis Uncovers Continuous Transitions Among Satellite Cell Subpopulations

**Figure 4:**
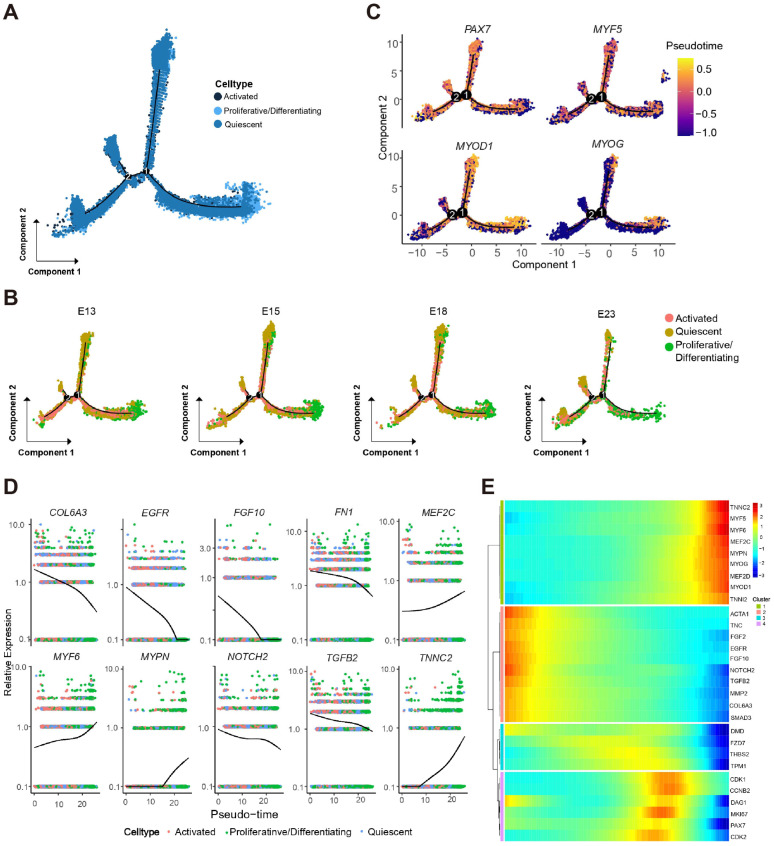
Single-cell trajectory analysis of satellite cell development: (**A**) Reconstruction of satellite cell developmental trajectories using Monocle 2, showing pseudotime progression. (**B**) Developmental trajectories of three satellite cell functional states (quiescent, activated, and proliferative/differentiating) across embryonic stages, with state transitions indicated. (**C**) Expression dynamics of key genes along the satellite cell developmental trajectory. (**D**) Pseudotime-dependent expression dynamics of key regulatory and recombination-related genes during SC development. (**E**) Pseudotime-resolved heatmap of dynamically expressed genes during SMSC development, revealing distinct gene expression modules associated with specific differentiation stages and cellular states.

To reconstruct the developmental dynamics of satellite cells, we conducted pseudotime trajectory analysis using Monocle2. The analysis revealed that satellite cells followed a sequential developmental continuum, transitioning from quiescent (Q-SC) to activated (A-SC) and ultimately to proliferative/differentiating (P-SC) states (Figure 4A). Early pseudotime points were predominantly occupied by Q-SCs, intermediate points by A-SCs, and later points by P-SCs, reflecting a clear progression. Branch point analysis showed a largely linear trajectory without major branching events, indicative of a unidirectional differentiation path. By mapping embryonic stages onto the pseudotime axis (Figure 4B), we observed that cells from early stages (E13 and E15) were enriched at early pseudotime points, while cells from later stages (E18 and E23) dominated the later pseudotime, further validating that pseudotime order mirrors the actual chronological sequence of embryonic development.

Dynamic gene expression analysis along pseudotime revealed regulatory patterns consistent with myogenic progression (Figure 4C): *PAX7* expression progressively decreased as cells advanced, whereas *MYF5* and *MYOD1* expression increased, corresponding to their respective roles in satellite cell activation and myogenic commitment. In contrast, *MYOG* expression remained low in quiescent and proliferative/differentiating states but rose sharply in the activated state, marking the onset of terminal differentiation.

Expression patterns of additional key myogenic regulators and structural components (Figure 4D,E)—including transcription factors (*MYOD1*, *MYOG*, *MYF5*, *MYF6*, *PAX7*, *PAX3*, *MEF2C*, and *MEF2D*), cytoskeletal and contractile proteins (*MYL4*, *ACTA1*, *TNNC2*, *MYPN*, *TNNI2*, *TPM1*, and *DMD*), cell cycle regulators (*MKI67*, *CDK1*, and *CCNB2*), extracellular matrix components (*COL6A3*, *FN1*, *TNC*, *MMP2*, *THBS2*, and *TIMP1*), and signaling factors (*EGFR*, *FGFR4*, *FGF2*, *FGF10*, *FZD7*, *TGFB2*, *SMAD3*, *NOTCH2*, and *JAG1*)—collectively supported a coordinated and continuous satellite cell progression through these states.

Together, these results demonstrate that satellite cell fate transitions in goose embryonic muscle development are continuous and ordered, driven by sequential shifts in cell state and regulatory gene expression.

### 3.5. Cell–Cell Communication Analysis Elucidates Signaling Networks Among Satellite Cell Subpopulations

**Figure 5:**
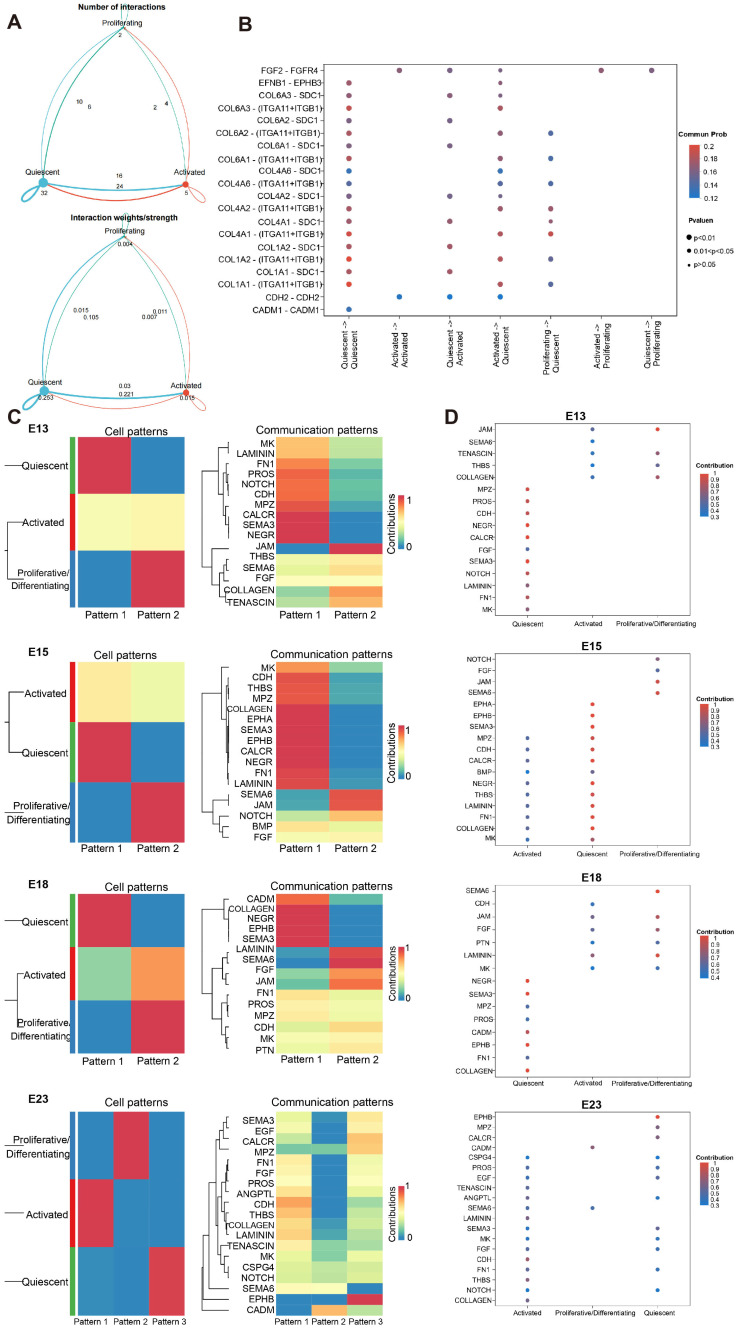
Cell–cell communication networks of skeletal muscle satellite cell (SMSC) populations: (**A**) Circle plots showing the number of interactions (**top**) and interaction strength (**bottom**) among proliferative/Differentiating, quiescent, and activated SMSCs. Nodes represent distinct cell types. The thickness of each line reflects the interaction strength be-tween cell types, and the line color corresponds to the color of the ligand-expressing cell type. (**B**) Key ligand–receptor pairs mediating cell–cell communication, including FGF2–FGFR4, EFNB1–EPHB3, and collagen–SDC1/integrin (e.g., COL6A1/2/3–SDC1 and COL6A1/2/3–(ITGA11 + ITGB1)), as well as homophilic CDH2–CDH2 and CADM1–CADM1 interactions. (**C**) Heatmap of incoming signaling pathway patterns across developmental stages (E13–E23), illustrating how target cells coordinate their responses to incoming signals. CellChat identifies global communication patterns (Patterns 1–3), with rows representing cell types (**left**) or signaling pathways (**right**), which were clustered into two major patterns. Red indicates higher pathway activity. (**D**) Bubble plot showing the contribution of each signaling pathway to the communication patterns of different functional states across developmental stages (E13–E23). Color intensity represents the contribution level.

To dissect the cell-state-specific communication landscape, we analyzed ligand–receptor interactions among quiescent, activated, and proliferative/differentiating satellite cell subpopulations. As shown in Figure 5A, quiescent satellite cells exhibited the highest number and strength of interactions, followed by activated cells, with proliferative/differentiating cells displaying the lowest communication capacity. This progressive decline suggests that satellite cells progressively reduce environmental interactivity as they transition toward commitment and differentiation, potentially facilitating autonomous myogenic programming. Figure 5B identifies key ligand–receptor pairs driving these interactions, including FGF2–FGFR4, EFNB1–EPHB3, multiple collagen–syndecan-1 (e.g., COL6A1/2/3/5–SDC1), and collagen–integrin complexes (e.g., COL6A1/2/3/5–(ITGA11+ITGB1)), as well as homophilic cell adhesion through CDH2–CDH2 and CADM1–CADM1. The prominence of ECM components and integrin-mediated contacts suggests dynamic extracellular remodeling during satellite cell state transitions, while FGF and Ephrin axes contribute to proliferation, migration, and spatial organization.

Subsequently, we systematically analyzed the signaling communication patterns of three satellite cell subpopulations across developmental stages (E13–E23). As shown in Figure 5C,D, the incoming signaling pathway patterns received by different cell types (quiescent, activated, and proliferative/differentiating) and the contribution of each signaling pathway to the communication patterns of distinct cell types differ considerably across developmental stages. CellChat analysis identified three global communication patterns (Patterns 1–3), with both cell types and signaling pathways clustering into two major patterns, in which red indicates higher pathway activity (Figure 5C). Figure 5D further quantified the contribution levels of each signaling pathway, revealing that the contribution intensities of pathways such as SEMA6, CDH, FGF, LAMININ, MK, MPZ, CADM, FN1, and COLLAGEN differed significantly among cell states. This indicates that satellite cells at distinct differentiation states exhibit state-specific responsiveness to microenvironmental cues.

Together, these findings demonstrate that during muscle development and regeneration, satellite cells dynamically regulate their signaling output and input, integrating extrinsic signals with intrinsic differentiation programs to coordinate orderly myogenic progression.

## 4. Discussion

Recent advances in single-cell transcriptomics have revolutionized our understanding of skeletal muscle development by revealing cellular heterogeneity and dynamic transcriptional landscapes across species, including pigs, cattle, chickens, ducks, and geese [12,14,15,37]. In this study, we performed 10× scRNA-seq to systematically map, for the first time, the cellular landscape of goose SMSCs during embryonic leg muscle development across four successive stages (E13, E15, E18, and E23). Compared to recent single-cell studies in avian species, our work offers several complementary strengths. Unlike Xu et al. (2025), who focused on the breast muscle of the Ding’an goose, and Sun et al. (2026), who examined duck leg muscle, our study provides a higher temporal resolution (four consecutive stages) specifically for goose leg muscle, enabling more precise tracking of cellular state transitions [14,15]. Additionally, while Li et al. (2025) compared two chicken breeds [11], our study presents a comprehensive atlas of all major cell types (satellite cells, myocytes, FAPs, endothelial cells, immune cells, and Schwann cells) in the goose, supported by the largest reported satellite cell dataset (23,587 cells) to date in waterfowl, which allows robust subclustering analysis. Together, these features position our work as a detailed single-cell resource for goose embryonic myogenesis, complementing existing studies in other avian species.

We identified six major cell populations—satellite cells, myocytes, FAPs, endothelial cells, immune cells, and Schwann cells—with satellite cells representing the largest proportion (55.7%), followed by myocytes (29.6%) and FAPs (13.2%). Although all six cell types were detected at each stage, their relative proportions changed dynamically, reflecting stage-specific cellular remodeling during embryonic myogenesis. These findings are largely consistent with the cell type succession patterns observed in mammalian myogenesis [38,39], supporting the conservation of core cellular mechanisms underlying skeletal muscle development between avian and mammalian species.

To further dissect the heterogeneity within the satellite cell population, we performed subclustering analysis on 23,587 satellite cells and resolved seven transcriptional subclusters that converged into three functionally distinct states: quiescent (Q-SC), activated (A-SC), and proliferative/differentiating (P-SC). This resolution provides single-cell-level evidence for the fate determination of satellite cells during goose embryonic myogenesis. Pseudotime trajectory analysis further confirmed a linear, unidirectional transition from Q-SCs to A-SCs and finally to P-SCs, without significant branching, indicating a continuous and ordered myogenic progression. Such linear trajectories have also been observed in developing mouse and human muscle, suggesting a conserved mode of satellite cell commitment [40,41]. The expression dynamics of *PAX7*, *MYF5*, and *MYOD1* across the three subpopulations followed a classical cascade: *PAX7* was broadly expressed in all subpopulations, *MYF5* was upregulated in A-SCs and P-SCs, whereas *MYOD1* was predominantly restricted to the P-SC subpopulation. This pattern is highly consistent with the established model of satellite cell activation and differentiation in mammals [42,43].

Differential expression analysis across the four embryonic stages identified 2603 consistently differentially expressed genes, which are involved in transcriptional regulation (*PAX7*, *MYOD1*, *MYOG*), sarcomeric assembly (*ACTC1*, *TPM1*, *TNNC2*), and cytoskeletal remodeling (*DMD*, *DYSF*, *SGCA*). GO and KEGG enrichment analyses revealed significant enrichment in pathways related to cell cycle regulation, metabolic reprogramming, Wnt signaling, and ECM–receptor interaction. These results are corroborated by our previous bulk RNA-seq study in ZW goose embryos, which identified 464 stage-shared DEGs exhibiting a temporal shift from structural assembly at E15 to ECM remodeling and lipid metabolism at E23, as well as a core regulatory module centered on *MEF2C*, *MEF2D*, *MYOD1*, and *MSTN* [9]. Collectively, these findings support a developmental progression from active myogenesis toward metabolic maturation and tissue stabilization, which aligns well with a recent scRNA-seq study in developing chicken pectoralis muscle that also identified a continuum of satellite cell states and highlighted the importance of ECM-related pathways [29].

In summary, our single-cell transcriptomic analysis reveals a temporally resolved and transcriptionally coordinated myogenic program in goose embryonic leg muscle. The identification of three functionally distinct satellite cell states—quiescent, activated, and proliferative/differentiating—along a linear pseudotime trajectory provides direct evidence for a conserved activation cascade in avian myogenesis. The dynamic shifts in cell population proportions and the stage-specific enrichment of pathways such as Wnt signaling, ECM–receptor interaction, and metabolic reprogramming collectively illustrate a developmental transition from active myoblast expansion to tissue maturation. These findings not only extend the current understanding of satellite cell heterogeneity in waterfowl but also establish a fundamental single-cell reference for comparing myogenic regulatory networks across avian species.

Despite presenting a comprehensive single-cell transcriptomic atlas of goose embryonic myogenesis, several limitations should be acknowledged. First, single-cell transcriptomic data alone cannot fully capture the spatial distribution of cells or the precise architecture of the stem cell niche. Future studies integrating spatial transcriptomics will be essential to validate cell–cell communication networks in situ and to map the physical proximity of satellite cells with myocytes and FAPs. Second, the key genes and signaling pathways identified here require further functional validation. Approaches such as CRISPR-Cas9 gene editing or small-molecule inhibitor treatment in in vitro or in vivo models are needed to confirm their precise roles in myogenesis. Third, our study focused exclusively on the leg muscle. Given the known heterogeneity of satellite cells across different anatomical locations (e.g., leg vs. breast muscle), future investigations should compare these trajectories to identify region-specific regulatory networks.

## 5. Conclusions

In summary, this study provides the first high-resolution single-cell transcriptomic atlas of goose embryonic SMSC development, revealing a linear trajectory of satellite cells from quiescent to activated to proliferative/differentiating states and key transcriptional regulators, including *PAX7*, *MYF5*, and *MYOD1*. These findings advance the fundamental understanding of avian myogenesis and offer a practical resource for improving muscle growth and meat production in waterfowl through targeted genetic selection, nutritional intervention, or cell-based agricultural strategies.

## Figures and Tables

**Table 1:** Summary of scRNA-seq read mapping and quantification.

Item	E13	E15	E18	E23	Aggregation
Estimated number of cells	16,077	11,781	11,527	11,261	12,955
Filtered number of cells	13,346	10,048	9780	9712	32,838
Mean reads per cell	25,553	29,116	30,580	35,810	30,648
Median genes per cell	3127	3551	3502	3964	3531
Valid barcodes	94.60%	94.60%	94.50%	92.30%	93.8%
Fraction reads in cells	96.60%	97.10%	96.40%	95.90%	96.3%
Reads mapped confidently to genome	86.30%	89.10%	87.90%	84.30%	86.17%
Reads mapped confidently to transcriptome	79.50%	81.90	81.90%	76.00%	79.13%
Number of reads	410,822,023	343,019,571	352,494,533	403,259,758	388,858,771
Total genes detected	21,551	20,885	20,802	21,193	21,182

## Data Availability

The original contributions presented in this study are included in the article/Supplementary Material. Further inquiries can be directed to the corresponding author(s). The raw and processed scRNA-seq data generated in this study have been deposited in the GEO database under accession number GSE328877.

## Supplementary Materials

The following supporting information can be downloaded at: https://www.mdpi.com/article/10.3390/cells15110983/s1, Figure S1: GO enrichment analysis of the 2603 core DEGs; Table S1: List of the 2603 DEGs consistently altered across all three pairwise comparisons. Table S2: GO enrichment analysis of the 2603 shared DEGs. Table S3: KEGG pathways related to the 2603 shared DEGs. Table S4: List of the 517 DEGs commonly shared across the three satellite cell functional states. Table S5: KEGG pathway analysis of the 517 shared DEGs identified in the three satellite cell functional states.
