# Supplementary figures and images for: Single-Cell RNA Sequencing Reveals Cellular Heterogeneity and Developmental Dynamics of Goose Satellite Cells During Embryogenesis
Source: Cells. 2026 May 27;15(11):983. doi: 10.3390/cells15110983 (PMC13256991; doi:10.3390/cells15110983)

# GO enrichment analysis(Co\_updown)

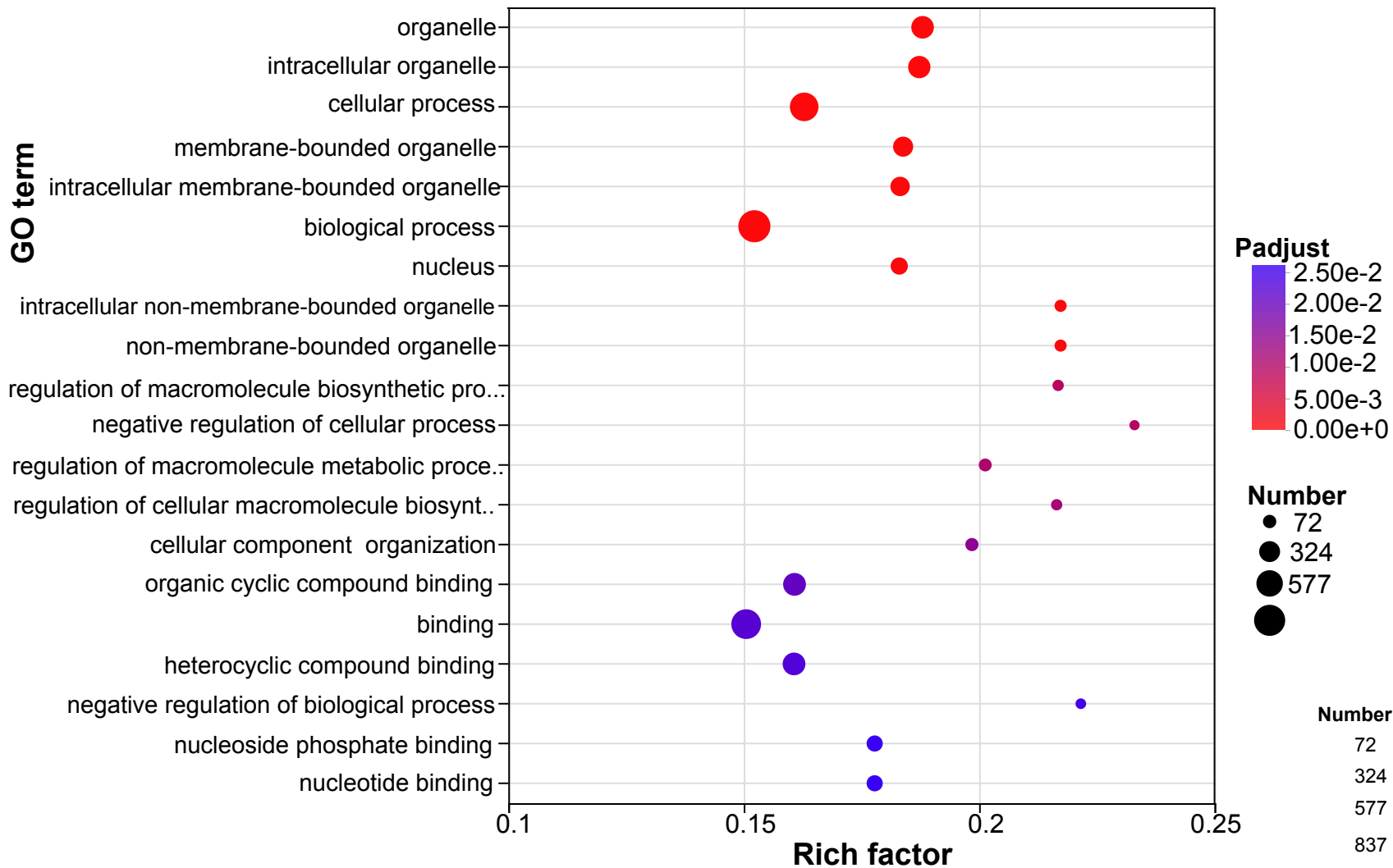

Supplement: Supplementary file 1 [file cells-15-00983-s001.zip › Figure S1.pdf]
